# Strategic planning as a catalyst for sustainability: A mediated model of strategic intent and formulation in manufacturing SMEs

**DOI:** 10.1371/journal.pone.0325887

**Published:** 2025-06-10

**Authors:** Ahmed Muneeb Mehta, Syeeduz Zafar Qazi, Rasheedul Haque, Abdul Rahman bin S Senathirajah, Waqas Baig, Rabia Sajjad, Dr. Abdul Rauf

**Affiliations:** 1 Hailey College of Banking and Finance, University of the Punjab, Lahore, Pakistan; 2 INTI International University, Nilai, Malaysia; 3 Wittenborg University of Applied Sciences, Apeldoorn, the Netherlands; 4 University of Business and Technology, Jeddah, Saudi Arabia; 5 Faculty of Business, Hospitality, Accounting and Finance (FBHAF), MAHSA University, Jenjarom, Malaysia; 6 Faculty of Business and Communications, INTI International University, Nilai, Malaysia,; 7 Wittenborg University of Applied Sciences, Netherlands; University of Central Punjab, PAKISTAN

## Abstract

This study examines the influence of Systematic Strategic Planning (SSP) on the Sustainable Performance (SP) of manufacturing Small and Medium Enterprises (SMEs) in Pakistan. Despite SMEs’ vital contribution to economic growth, there is limited empirical research on how strategic planning enhances sustainable performance in SMEs operating in emerging economies facing political and economic instability. Drawing on the Triple Bottom Line (TBL) and Resource-Based View (RBV) theories, this study investigates the mediating roles of Strategic Intent (SI) and Strategic Formulation (SF) in the SSP-SP relationship. A quantitative research design was employed, and data were collected through structured questionnaires distributed to senior executives and decision-makers of manufacturing SMEs. A total of 410 valid responses were received. Structural Equation Modeling (SEM) was applied using AMOS 28 software to analyze the data and test the hypothesized relationships. The results demonstrate that SSP has a significant direct effect on SP and an indirect effect through SI and SF. Specifically, the components of SSP—strategic analysis, strategy creation, strategy execution, and monitoring and evaluation—enhance SMEs’ economic, environmental, and social performance. The study highlights that adopting systematic strategic planning practices enables SMEs to navigate complex and uncertain environments, achieve competitive advantage, and contribute to sustainable development goals. This research fills a critical gap in the literature by focusing on manufacturing SMEs in Pakistan, an under-researched context in the sustainability and strategic management fields. It offers practical insights for SME managers and policymakers to develop and implement comprehensive strategic planning frameworks that foster sustainability. The study also provides theoretical contributions by integrating SI and SF as key mediators within the TBL and RBV theoretical frameworks.

## 1. Introduction

Small and Medium Enterprises (SMEs) play a significant role in driving economic development, employment generation, and innovation globally. In emerging economies such as Pakistan, manufacturing SMEs contribute substantially to industrial output and economic growth. However, these enterprises often operate in environments marked by political instability, economic volatility, and resource constraints, making sustainable performance (SP) difficult to achieve. The need for effective strategic planning is increasingly critical as SMEs strive to ensure long-term survival and competitiveness under such challenging circumstances.

Strategic planning has been recognized as an essential tool for organizations to navigate uncertain environments, allocate resources efficiently, and achieve strategic goals. While prior studies have explored the relationship between strategic planning and organizational performance, there is limited empirical research investigating how systematic strategic planning (SSP) contributes to sustainable performance, particularly in SMEs operating within emerging economies. Furthermore, existing literature has predominantly focused on large organizations in stable contexts, neglecting the unique challenges faced by SMEs in less predictable settings.

This study addresses this gap by examining the influence of SSP on the sustainable performance of manufacturing SMEs in Pakistan. Drawing on the Triple Bottom Line (TBL) and Resource-Based View (RBV) theories, this research introduces Strategic Intent (SI) and Strategic Formulation (SF) as mediating variables that explain the mechanisms through which SSP enhances sustainable performance. By integrating these constructs, the study offers a comprehensive theoretical framework that advances understanding of strategic management practices aimed at achieving sustainability in SMEs.

The emergence of globalization has significantly altered corporate operations; consumer demands and expectations are changing, rivalry is escalating, and sophisticated technologies are being employed in the industry. The surge in energy prices stemming from the COVID-19 epidemic and the conflict in Ukraine has intensified the global energy crisis, leading to a significant increase in power procurement costs and compelling governments and corporations to explore alternate energy sources, such as wind energy. As a result, enterprises struggle to maintain growth and a competitive advantage in the rapidly changing economic environment. Therefore, a company’s strategy must be developed and modified as needed to sustain long-term competitiveness. Thus, enterprises that deliver authentic superior value to the customer experience via an innovative corporate strategy rooted on long-term viability and sustainability will endure and prosper throughout time [1]. The COVID-19 pandemic has resulted in the closure of thousands of SMEs worldwide and necessitated a reorganization of the global economy. Therefore, several firms, especially SMEs, are encouraged to adopt new business models with strong and creative strategies to attain sustainability [2].

Similarly, sustainability has become a problem in modern production industries because to the limitations of conventional manufacturing techniques and the requirements enforced by stakeholders. Sustainable manufacturing entails the creation of cost-effective goods that need little resources, demonstrate minimal environmental impact, and guarantee societal safety [3]. Thus, the adoption of sustainability concepts by manufacturing SMEs has become a priority to tackle the aforementioned challenges.

The manufacturing SME sector in Pakistan has several challenges and barriers that hinder its growth and limit its ability to attain a higher level of sustainable performance. The impediments can be categorized into three principal types: significant, structural, and internal. The principal issue is external, arising from political turmoil that obstructs economic advancement in Pakistan [4]. The second obstacle is internal and recognized as a structural hindrance stemming from the insufficient involvement of Pakistani policy in controlling, regulating, and fostering a favorable climate for the private sector and manufacturing industry to prosper. The third principal hurdle, which will be the emphasis of this research, is to internal problems at the company level, arising from inadequacies in management best practices, particularly the implementation of effective strategic planning and business innovation [5]. Furthermore, the majority of Pakistani SMEs are family-owned businesses facing considerable challenges to their survival and sustainability, resulting in the insolvency of several SMEs [6–10]. Prior research on the Pakistani manufacturing sector reveals that these challenges may be summarized by the fact that the market share of locally manufactured items in the domestic market does not exceed 20 percent. The manufacturing SMEs in Pakistan do not fully utilize their potential. They are functioning at only 50 percent of their production capacity, and elevating it to 70 percent will yield around 40,000 more jobs [11–13]. As a result, manufacturing SMEs in Pakistan encounter significant competition from imported products, resulting in a trade deficit of USD 5.4 billion [14]. Furthermore, most SMEs in the country have significant challenges that jeopardize their sustainability, mostly due to inadequate institutionalization at the organizational level. They have structural management challenges and export limitations [15,16].

The aforementioned factors hinder the capacity of Pakistani SMEs to expand, compete, and maintain viability. These challenges significantly impact the efficiency and sustainability of SMEs. This study posits that resolving the identified internal issues and achieving enhanced sustainable performance across economic, environmental, and social dimensions for manufacturing SMEs in Pakistan can be accomplished through the effective implementation of systematic strategic planning, encompassing strategic intent and formulation. Consequently, many challenges faced by SMEs in Pakistan may be categorized and addressed through these elements: systematic strategic planning with defined objectives and strategy development [17].

Typically, SMEs function with less formality; hence, the components of systematic strategic planning and innovation may not be well addressed. Limited empirical research has established a correlation between strategic planning attributes, business innovation, and the enhanced performance of SMEs, contributing to their sustainability [13,18]. This study aims to evaluate the influence of systematic strategic planning (SSP) and strategic formulation (SBI) on the sustainable performance of manufacturing SMEs in Pakistan.

This research makes several key contributions. First, it provides empirical evidence from Pakistan’s manufacturing SME sector, a context that remains under-explored in sustainability and strategic management literature. Second, the study introduces an innovative framework by combining TBL and RBV theories with the mediating roles of SI and SF. This approach extends theoretical understanding of how SSP can foster economic, environmental, and social sustainability in SMEs. Finally, the findings offer actionable insights for managers and policymakers seeking to implement effective strategic planning processes to promote sustainability in resource-constrained and volatile environments. The subsequent sections of the paper are structured as follows. Section 2 offers a theoretical analysis of the literature, including theories, variables, and concepts. Section 3 provides a comprehensive review of the methodologies utilized for data gathering and analysis. Section 4 delineates the results of the inquiry. Section 5 analyzes the study’s results in comparison to previous research. Section 6 delineates the results and recommendations.

## 2. Literature review

This section provides a theoretical analysis of the ideas, concepts, and variables utilized in this study. The aim is to develop a theoretical model and hypothesis.

The literature has focused on the impact of strategic planning and business innovation on sustainable performance in large businesses. The viewpoint of SMEs is significantly neglected, especially in developing countries. Small and medium-sized firms (SMEs) are crucial to the success of economic growth cycles in industrialized, rising, and developing nations, since they serve as a primary source of employment creation. Governments in emerging nations significantly depend on the effectiveness of SMEs to create employment, unlike established economies that primarily rely on the success of large local and international firms [19]. The overall economic stability of a nation may decline if SMEs do not possess a coherent plan and adequate financial and non-financial resources, including suitable human capital and affordable raw materials and energy. Therefore, authorities and support organizations must provide extensive help to SMEs, including policy support and legislation that promote a favorable business climate [10,20]. Despite their substantial contribution to global economic growth, SMEs face challenges associated with material scarcity, as their raw material consumption is anticipated to quadruple by 2060 due to global economic development and an enhancement in average quality of life [21]. Therefore, SMEs must adeptly adjust their operations to conform to the sustainability model, since several businesses frequently face obstacles pertaining to sustainable growth [8,22]. They must prepare to address the expected scarcity of raw materials by enhancing their resource efficiency, energy efficiency, and production techniques [23–25]. Concurrently, industrial enterprises must comply with rigorous environmental rules and heightened societal pressure stemming from deteriorating climatic circumstances [10,25,26]. Therefore, all businesses, especially SMEs, are encouraged to adopt innovative business models employing resilient and creative strategies to attain sustainability [8,27].

In Arab nations, small and medium-sized firms (SMEs) propel economic growth by creating job opportunities and promoting innovation [24]. Nevertheless, SMEs in emerging economies have managerial issues that hinder their growth, reduce their expected performance, and threaten their sustainability. The challenges can be summarized as follows: first, impediments to long-term planning; second, a deficiency in vision and strategic direction; third, inadequate planning capabilities; fourth, a lack of creative and innovative solutions to adapt to evolving business environments; fifth, insufficient resilience to respond to changing conditions; sixth, heightened operational costs; and seventh, a reduced ability to articulate specific challenges and delineate constraints [28]. Moreover, the senior management of SMEs in developing countries has a deficiency in business acumen [8,9,29].

Scholars and experts worldwide have endorsed improved strategic planning and resilience in small and medium-sized organizations (SMEs). They have underscored the need of implementing strategic physical and intangible enablers, including business innovation. Numerous studies have demonstrated that systematic strategic planning (SSP) may aid organizations of any size in improving their economic, environmental, and social performance, either individually or collectively [30]. However, scant study has investigated the cumulative effect of the aforementioned strategic factors on the sustainable performance of firms in the manufacturing sector, especially SMEs, in developing countries. This study will investigate the impact of strategic factors, SSP, and the mediating effects of SI and SF on the sustainable performance (SP) of manufacturing SMEs in Pakistan within a highly volatile environment.

### 2.1. Triple bottom line (TBL) and Resources Based View (RBV) theories

The research model of this study is founded on the ideas of the triple bottom line (TBL) and the resource-based perspective (RBV). Sustainability and the triple bottom line (TBL) are two interconnected ideas frequently cited in the literature [29,30]. Furthermore, the Triple Bottom Line (TBL) offers a framework for evaluating the performance and achievements of corporate entities across three equitable dimensions: economic, social, and environmental. Consequently, the acronym TBL has been employed to signify the operational basis for sustainability [31]. During the early 1990s, the word TBL was not widely recognized. The term TBL was established in the 1990s by John Elkington, a business development consultant; it encompasses the economic, environmental, and social worth of assets that may extend beyond a firm’s financial bottom line. Elkington defined the Triple Bottom Line (TBL) via the aspects of profit, people, and planet. The TBL assigns equal importance to each of the three dimensions, hence improving the construct’s balance and coherence [32]. The TBL approach exploits value qualities with more accuracy and utilizes available tangible and intangible resources more efficiently. Consequently, firms’ assets must be utilized properly and efficiently. As a result, firms are increasingly evaluated according to their environmental impacts on stakeholders and their substantial economic and social performance results. Sustainable performance signifies that firms may achieve favorable social, environmental, and economic results, known as the “People, Planet, and Profit 3Ps,” which constitute a triadic, value-enhancing framework. Thus, sustainability management has three equally important dimensions: environmental, social, and economic.

The evaluation of corporate performance and strategic management is essentially grounded on several core concepts, including the Resource-Based View (RBV) [12,13,24,33,34]. [35] posited that each business had unique intangible and tangible assets, which vary in capabilities, competitive advantages, skills, competences, experience, methodologies, procedures, and information systems. Thus, the Resource-Based View (RBV) emphasizes the significance of a business enterprise’s competitive advantages and capabilities for its sustainability [36]. A corporation that upholds quality, originality, uniqueness, and non-substitutability can achieve enduring competitiveness. The Resource-Based View (RBV) emphasizes the importance of a company’s tangible and intangible resources, together with human talents, illustrating the uniqueness of these assets that facilitate sustained success for enterprises [37,38]. A review article investigating the application of Resource-Based View (RBV) theory to a firm in empirical research highlighted that the RBV has been thoroughly examined as a framework for comprehending the determinants that facilitate a company in attaining a sustained competitive advantage [39]. By employing the triple bottom line (TBL) and Resource-based view (RBV) theories, businesses, especially SMEs, can improve their understanding of maintaining business performance and developing a competitive advantage in a volatile environment through the effective utilization of their capabilities and resources. This research study utilized the TBT and RBV theories to analyze the sustainable performance of manufacturing SMEs in Pakistan, concentrating on their environmental, economic, and social results.

### 2.2. Sustainable performance

Recent trends in economic and social growth have significantly escalated the consumption of products, leading to the depletion of natural resources and perhaps jeopardizing the health of human populations [40]. The assessment of corporate performance is evolving to incorporate social and environmental factors alongside financial outcomes [16,41]. Recently, the majority of business owners, CEOs, and experts have evaluated firm success solely by financial metrics such as revenue, profitability, and market share, neglecting additional intangible indications [10,42]. Consequently, the assessment of solely physical performance indicators in firms has proven inadequate for dynamic competition, has constrained natural and skilled human resources, and has exacerbated environmental limits [9,13]. Therefore, businesses must increasingly incorporate sustainable performance metrics, including community satisfaction and ecological indicators, in conjunction with traditional financial measurements of success. Recently, private enterprises have initiated proactive measures to reconcile their economic viability with environmental and social performance, aiming for enhanced long-term sustainability, indicative of a favorable transformation [38,43].

In early 1969, the American National Environmental Policy Act (NEPA) articulated a growing concern over the vital interplay between humans and the environment in response to these concerns [8,10,44]. In 1987, the United Nations Brundtland Commission promoted the global acceptance of these ideas. It provided the authoritative and most commonly used definition of sustainable development: “meeting the needs of the present without compromising the ability of future generations to meet their own needs” [25,45]. Sustainability has evolved into a multifaceted concept, with diverse meanings contingent upon the specific topic and academic subject. Consequently, sustainability is a multifaceted notion that includes economic, social, political, technological, and environmental factors. Since the Brundtland Report (1987), most researchers concur that sustainability may be categorized into three domains: economic, social, and environmental, emphasizing the equilibrium among them within the corporate framework [46,47]. Sustainability plans emphasize company development and superior performance for the advantage of the public, society, and the environment [48–50]. Previous studies indicate that social, environmental, and economic performance must be evaluated simultaneously as an integrated package, rather than in isolation, to enhance business operations, foster enterprise performance, promote social harmony, and safeguard the environment [21]. As sustainable development gains prominence, businesses must proactively plan and articulate the beneficial impact of their activities on the environment and community [28].

The economic performance of a firm is defined as its ability to achieve short, medium, and long-term objectives financially. Furthermore, the usual approach to assessing a company’s economic performance involves monitoring standard indicators such as return on equity (ROE), return on assets (ROA), return on capital employed (ROCE), and return on sales (ROS) [10,51,52]. The principal factors influencing economic sustainability in commercial enterprises, particularly SMEs, are substantial savings, elevated profitability, first-mover advantage, and robust competitive advantages [25,53]. Economic performance pertains to company strategies that address market expectations by delivering products promptly, efficiently, and profitably to sustain or enhance overall quality of life [54–56].

Environmental performance often denotes a corporation’s initiatives to cultivate sustainable practices and promote environmental conservation, encompassing the preservation of ecosystems and the environment. Consequently, environmental conservation seeks to preserve the natural ecosystem, including individual organisms and their life support systems [7]. Furthermore, the ecologically sustainable growth of manufacturing SMEs and the implementation of environmentally friendly initiatives are crucial for sustaining industrial competitiveness and sustainability. Moreover, in the industrial sector, environmental performance pertains to actions that utilize natural resources to mitigate environmentally detrimental impacts [57].

The environmental performance of an industrial business may be assessed by its ability to respond to, control, and mitigate emissions of CO2, solid waste, water usage, and energy consumption. It also encompasses the firm’s commitment to reducing the utilization of hazardous, unsecure, and toxic items while mitigating the incidence of environmentally detrimental mishaps [49,50,58]. Subsequently, many measures have been implemented, including a reduction in energy, power, and resource consumption; a decrease in pollution and waste; compliance with environmental regulations; and enhancement of the firm’s environmental image, all of which are purported to be advantageous [20,56]. Small and medium-sized enterprises (SMEs) should also examine other environmental performance factors such as ecology, adherence to national and international environmental standards, and other critical information, including environmental expenditures and the ecological impacts of products and services. The dedication of top management and the unique attributes of senior executives will be crucial for the integration of environmental standards in SMEs. Consequently, companies may establish credibility and get financial and non-financial resources by adopting and integrating elements of environmentally sustainable performance [59].

Alongside the economic and environmental aspects of sustainability, social responsibility constitutes the third essential component that has been comparatively underrepresented in the literature; thus, the interconnectedness of the environmental, economic, and social dimensions is vital to sustainability across developing, emerging, and developed nations [60]. As customers increasingly demand that firms exhibit compliance with social responsibility, it is no longer adequate to achieve only financial and environmental outcomes. Consequently, effectively managing social performance alongside economic and environmental performance is essential for managers, who must be knowledgeable about strategies to proficiently oversee social performance [61]. Social sustainability, while far more challenging to assess than economic growth or environmental impacts, is the most neglected component of the triple bottom line in comparison to economic and environmental performance. Likewise, the majority of social sustainability indicators are excessively broad to be useful, necessitating the development of tailored metrics for specific organizations. The pursuit of sustainability, particularly social sustainability, has commenced reshaping the competitive marketplace, compelling organizations to reevaluate their strategies regarding products, technology, processes, and business models owing to this transition [5,16]. Consequently, the principal objective beyond social responsibility is the preservation and conservation of communal cohesiveness. Consequently, social performance is linked to the business strategies that affect the fundamental requirements of the company’s investors, employees, communities, and society at large [62].

Multiple-research in management literature have examined the determinants of financial performance. Nonetheless, limited research has investigated how manufacturing SMEs might develop and execute long-term plans to address sustainable performance concerns [13]. Likewise, further studies are necessary, particularly empirical study in small and medium-sized enterprises (SMEs). Consequently, more research is advised to develop a standard integration of the three primary aspects of corporate sustainability via the organization’s aims, competitive advantages, and internal/external legality [38,63]. The focus on examining sustainable performance in commercial enterprises, particularly small and medium-sized enterprises (SMEs), has progressively expanded in recent years in both established and emerging nations. This indicator requires more research across many business sectors in emerging nations. The expansion of research on sustainable performance in SMEs is ascribed to two primary factors: Small and medium-sized enterprises (SMEs) play essential and dynamic roles in the economies of emerging nations. Consequently, SMEs must address internal and external factors that affect financial, social, and environmental outcomes [46,56]. The second reason is the crucial involvement of SMEs in achieving the sustainable development goals (SDGs) at the national level [64].

It is essential to examine how elements like systematic strategic planning with strategic intent and strategy formulation influence sustainable performance in a developing nation characterized by a volatile environment and suboptimal firm performance, such as Pakistan [40]. This research defines sustainable performance as a business’s capacity to integrate social, environmental, and economic aspects [65]. Consequently, social, environmental, and economic performance are evaluated simultaneously as an integrated entity rather than in isolation, serving as a strategy for profit generation, enhancing enterprise performance, fostering social cohesion, and safeguarding the environment [16,66,67]. This approach may facilitate the identification of pragmatic recommendations and theoretical frameworks that support the growth, sustainability, and longevity of SMEs.

### 2.3. Systematic strategic planning (SSP)

Strategic planning is quite appealing as a study domain across many businesses. It has been widely utilized across many industries, including manufacturing and service enterprises, as well as in governmental and non-governmental sectors, ultimately establishing itself as a fundamental pillar of management [68]. In a changing business environment, the ability of enterprises to adapt rapidly and efficiently is a crucial success component that necessitates a well-defined plan. Consequently, systematic strategy planning has evolved to the extent that its primary value is in aiding businesses to operate effectively within a dynamic and complex environment while navigating more uncertain conditions [38,69]. Systematic strategic planning, or formal strategic planning, comprises a sequence of interconnected processes, including strategy formulation, whereby internal and external factors undergo meticulous analysis and evaluation. This process encompasses the strategy’s formulation phase (comprising the mission, vision, strategic objectives, plans, and strategic alternatives), the implementation phase (the execution of the strategy), and subsequently the mentorship and evaluation stages [70].

Systematic strategic planning allows organizations to enhance their value and fortify, uncover, and dominate their competitive market position. Furthermore, it provides leaders with appropriate strategies and measures necessary for achieving enduring competitive advantages [70]. Similarly, all types of companies are urged to establish strategic plans that enable them to compete and endure; therefore, senior management must embrace and devise strategies utilizing contemporary instruments to get superior outcomes. Furthermore, organizations are encouraged to regularly assess the internal and external environment and adjust their strategies appropriately, alongside undertaking monitoring and assessment, both of which are crucial for the strategic planning process [71,72]. Consequently, companies achieve superior performance and maintain sustainable outcomes across financial, operational, and non-financial dimensions when strategic planning is formally and officially executed [73,74].

Despite extensive study in this field, significant gaps remain in the literature, particularly with the analysis of integrative models of the systematic strategy process, which should encompass all stages as a cohesive unit in relation to sustainable performance. The research undertaken in impoverished nations is restricted, inadequate, inconclusive, and requires more investigation [75]. This research aims to evaluate the impact of systematic strategic planning on the sustainable performance of manufacturing SMEs in Pakistan, with the objective of formulating recommendations and guidelines to improve the systematic strategic planning process, thereby enhancing the performance and sustainability of these SMEs, which currently face numerous challenges threatening their viability.

### 2.4. Strategic planning and sustainable performance

Strategic planning is widely acknowledged as a critical process for organizations aiming to enhance performance and achieve long-term objectives. For SMEs, particularly in emerging economies, systematic strategic planning (SSP) is a vital mechanism to manage uncertainty, allocate resources effectively, and enhance competitiveness. Prior research suggests that SSP can lead to improved organizational outcomes, including financial stability and operational efficiency [15,43]. However, much of the existing literature focuses on large firms in stable contexts, with limited exploration of how SSP impacts sustainable performance (SP) in SMEs operating in volatile environments [62].

Sustainable performance, in this study, encompasses economic, environmental, and social dimensions, following the principles of the Triple Bottom Line (TBL) framework [18]. While several studies affirm that strategic planning can enhance financial outcomes, there is limited empirical evidence linking SSP directly to environmental and social sustainability, especially in SMEs within emerging markets.

### 2.5. Strategic intent and strategic formulation

Strategy intent and strategy formulation are essential elements of systematic strategic planning. Strategic purpose embodies an organization’s long-term ambitions and dedication to realizing its objectives, acting as a guiding principle for decision-making and resource distribution [76]. Manufacturing SMEs that express a distinct strategic intent are more capable of aligning their operations with overarching company goals, hence guaranteeing coherence across diverse functional areas [77]. Strategic formulation entails the creation of implementable strategies to achieve strategic objectives. It includes environmental scanning, SWOT analysis, competitive positioning, and resource prioritization [57]. In the SME manufacturing industry, strategic formulation is essential for identifying growth possibilities, reducing risks, and guaranteeing optimal resource allocation.

a) **Mediating Role of Strategic Intent and Formulation**

Strategic Intent (SI) reflects an organization’s long-term vision and commitment to achieving strategic objectives, often under conditions of uncertainty [54]. Strategic Formulation (SF), on the other hand, involves developing concrete plans and policies to realize strategic goals. Existing studies often treat these components as inherent parts of strategic planning [70], but their mediating roles in the SSP-SP relationship remain under-explored.

This study addresses this gap by conceptualizing SI and SF as key mediators. SI drives organizational focus toward sustainability objectives, while SF operationalizes strategic planning into actionable initiatives that contribute to economic, environmental, and social outcomes. By examining these mediators, this study offers a more nuanced understanding of how SSP translates into sustainable performance.

The mediating role of strategic aim and formulation in linking systematic strategic planning to sustainable performance is an emerging area of research. Strategic purpose acts as a link between planning and execution by fostering a shared understanding of organizational goals across all levels of the company [78]. Concurrently, strategic formulation transforms this desire into actionable steps, ensuring that strategic objectives are systematically pursued. For manufacturing SMEs, these intermediaries enhance the effective transformation of strategic plans into operational practices. Empirical evidence demonstrates that companies with a strong strategic purpose and clear strategies achieve higher levels of innovation, productivity, and sustainability [79]. Furthermore, these mediators enhance the advantageous outcomes of systematic strategic planning by refining organizational focus, reducing waste, and fostering a culture of continuous improvement [75].

b) **Strategic Planning in SMEs: Global and Local Perspectives**

Strategic planning in small and medium-sized enterprises (SMEs) is a crucial intervention to increase organization resilience, performance and sustainability. More broadly, many authors across the world have argued that SMEs should include strategic planning to survive the peaks and troughs of their markets [34]. Large corporations may possess resources and systems to resist disruptions in the environment, but SMEs are also more vulnerable, given limited financial and human capital [53].

The SME’s in Pakistan account for almost 90% of all enterprises and contribute almost 40% of GDP (SMEDA, 2023). Nevertheless, usually small and medium-sized enterprises do not have a formal strategic planning process in place due to lack of expertise, short-term focus and informal decision-making culture [67]. In the past, failure of formal planning has been linked with poor market responsiveness, loss of opportunities for innovation, economic shock vulnerability [70]. Therefore, systematic strategic planning (SSP) is no longer only a managerial tool but a strategic necessity to effectively survive and compete in developing economies.

c) **Strategic Intent as a Driver of Organizational Alignment**

Strategic intent is the goal of an organization and the commitment to attain the goal indefinitely in light of their limited resources, often because of limited resources [62]. In small and medium-sized enterprises where resource constraints are a longstanding problem, establishing strategic intent acts as a line of inquiry for organizational actions that align with the organizational long-term goals. Strategic intent that is coherent promotes cohesion among various departments, promotes innovation, and provides direction for decision making [19].

In manufacturing (SMEs), strategic intent might mean integrating both cost-effective (reduced in production cost) as well as environmental demands at the same time. Elucidating the impact of strategic intent in decision making is well-established by [57] who argue that strategic intention shapes firm responses to market disruptions as well as sustainability policy direction in small and medium-sized enterprises (SME). Strategic intent can mitigate systemic instability in emerging economies such as Pakistan, by incorporating resilience and anticipatory thinking aspects of business processes [46].

d) **Strategy Formulation: Bridging Vision and Action**

Strategy formulation is the process by which organizations formulate concrete plans in order to achieve strategic objectives through environmental scanning, SWOT analysis, capability assessment and policy development [71]. In SMEs this stage is usually informal or ad hoc but literature indicates that structured strategy formulation can have a positive impact in terms of visualizing the vision of an organization and turning it into practical initiatives [21,36,50].

Evidence also suggests that informal formulation of strategies of SMEs leads to the better allocation of resources, improving risk management and empowerment of innovation [51,66]. For the SMEs in Pakistan that make use of formal formulating processes and are well-structured these issues will be addressed which can help overcome operational inertia and external environmental volatility. [38].

e) **Integrative Models of Strategic Planning and Sustainability**

While traditional studies typically included evaluation of strategy and performance in isolation, contemporary scholarship advocates for integrative models that account for mediator mechanisms that complement them, such as strategic intent and strategic formulation [9,15]. Since these mediators exist, researchers will be able to learn not only whether strategic planning works, but also how it works to maximize sustainability.

As recent literature has highlighted, sustainability can best be achieved through strategic planning organized within the triple bottom line (TBL) perspective–i. e., considering the economic, environmental and social axes [49], and through the resource-based view (RBV), which is a theoretical framework that outlines how specific firm resources such as strategic intent, innovation capability and strategic formulation can be used as sources of sustained competitive advantage [31,67].

This mediated model has been applied to several sectors and regions, but research on the effects of this mediated model remains sparse in developing countries. As illustrated by Afum et al. (2021), in Ghanaian SMEs, strategic intent significantly increased the consequences of planning on sustainability. Also as illustrated by [66] in Greek SMEs, strategy formulation mediated the relationship between planning and innovation outcomes.

f) **Strategic Planning and Sustainability in the Pakistani Context**

Through a special socio-political and economic context Pakistan’s SME sector has an extraordinarily strong position – both politically and economically, where uncertainty about policies, unfavorable state of infrastructure and global financial stress [11]. Thus, sustainability is no longer just an option but a way of life. Although there is evidence to indicate that Pakistani SMEs are aware and practice sustainability strategies well above the recommended threshold [54,70].

In this context the proposed model focusing on SSP, SI and SF can serve as a roadmap for SMEs in attaining strategic alignment and long-term sustainability. Further, embedding sustainability issues into the strategic DNA of SMEs could lead to enhanced stakeholder trust, market reputation, and operational resilience in Pakistan [18,24].

The conclusion thus drawn is that SSP + mediating variables like SI and SF provide a theoretically robust and feasible framework for solving various performance and sustainability problems faced by the manufacturing SMEs in Pakistan. Further empirical work in this direction would facilitate validation of the model and policy makers would be able to formulate evidence-based strategies to support sustainable transformation of the sector.

### 2.6. Hypotheses development

Despite the growing body of literature on strategic planning, several limitations persist. First, there is a lack of empirical research focusing on SMEs in emerging economies, where the dynamics of political and economic instability present unique challenges [43]. Second, while TBL and RBV theories have been used extensively to examine sustainability and competitive advantage, few studies have integrated these frameworks to explore the indirect mechanisms through which SSP influences SP. Additionally, previous research has often overlooked the distinct characteristics of SMEs, such as limited resources and informal decision-making processes, which can affect the adoption and implementation of strategic planning for sustainability [67]. This study seeks to fill these gaps by developing a comprehensive framework that incorporates both SI and SF as mediators within the SSP-SP relationship.

This study’s research model and hypotheses were developed and validated by an extensive literature review to assess the effectiveness of the variables: systematic strategic planning, strategic intent, and strategic formulation. This study investigates the correlation between these attributes and the sustainable performance of the SME manufacturing sector in Pakistan. Systematic strategic planning was selected as an independent variable for several reasons. [80], in a literature review of more than thirty relevant empirical studies, recommend more study to explore the direct influence of strategic-planning formality on organizational effectiveness, which aids in sustainability [29]. Secondly, most prior research investigating the effects of systematic strategic planning on the performance and sustainability of business enterprises has been conducted in the US, Western, and emerging nations, with limited studies in developing countries, especially Arab nations [69].

Thirdly, studies on the effects of comprehensive strategic planning on performance have primarily concentrated on large businesses, particularly in the IT, electronics, and consulting industries. However, limited research has been undertaken on small and medium-sized firms, especially manufacturing SMEs, in Pakistan [81]. Pakistani manufacturing SMEs are prominent importers of raw materials and substantial job generators. Therefore, this contextual gap requires more investigation. Ultimately, the successful strategies derived from suitable SSP are seen as an intangible asset that may provide a competitive advantage to enterprises [82]. Therefore, more empirical research is necessary in Pakistan to corroborate previous results about the correlation between systematic strategic planning and sustained performance. Moreover, the unique settings of Pakistanis, their commercial activities, and cultural traits may enable the discovery of new insights concerning this connection. The study’s hypothesis on systematic strategic planning is as follows.

**Hypothesis 1 (H1). Systematic strategic planning enhances sustained performance in manufacturing SMEs in Pakistan.**

Conversely, Pakistan is not recognized as creative on the Global Innovation Index [83]. Similarly, based on the World Bank’s business performance rating, it is deemed an unattractive nation for foreign investors [84] Thus, the study investigates the mediating functions of strategic purpose and strategic formulation, providing a framework for understanding these components in various ways. This may present many policy recommendations to support manufacturing SMEs in Pakistan. In conclusion, previous research on strategic purpose and strategy creation has shown several reasons for adopting these elements as mediators. [85] propose examining the influence of strategic intent on the performance of Arab manufacturing SMEs to formulate recommendations for enhancing performance at both policy and corporate levels [86]. Secondly, most prior studies focused on analyzing the connections between the execution of strategic intent, strategic formulation, and traditional financial performance, e.g., [73,74], In contrast, this study will expand the performance concept by investigating the influence of implementing strategic intent and strategic formulation on sustainable performance as mediators. The bulk of strategic business innovation research has mostly been conducted in established and emerging nations, with a scant number of studies carried out in Arab countries, which are largely considered less developed [32]. Therefore, the hypothesis about mediating variables in this study is as follows.

**Hypothesis 2 (H2). Strategic intent mediates the connection between systematic strategic planning and sustained performance in manufacturing SMEs in Pakistan.**

**Hypothesis 3 (H3). Strategic formulation mediates the connection between systematic strategic planning and sustained performance in manufacturing SMEs in Pakistan.**

The framework postulates that while SSP has a measurable direct impact on SP, a considerable portion of its influence operates indirectly through SI and SF. This aligns with resource-based and capability-building views, suggesting that structured strategic actions lead to sustainability only when supported by intent and formulation mechanisms.

This study use uni-item measures to assess the four theoretical constructs: SP, SSP, SI, and SF. The SP scale, derived from [87] and [88], is assessed using nine items. The SSP scale, derived from [89], is assessed using seven items. The SI scale, derived from sources [90], is assessed using four elements. The SF scale, derived from sources [90], is assessed using three elements. These items were utilized to assess each construct in the identical manner as employed by the aforementioned prior research for the same structures. They were furthermore utilized for collecting data from similar respondents inside a small and medium-sized firm context.

**Fig 1:**
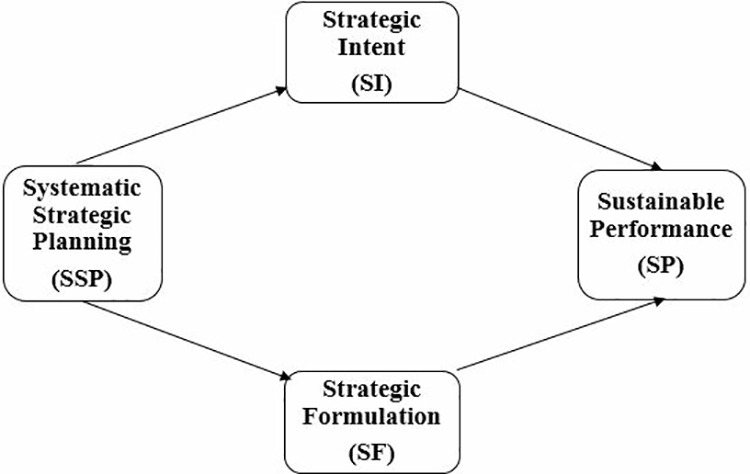
Theoretical Framework Illustrates the relationship between SSP, SI, SF, and SP in manufacturing SMEs.

The theoretical framework of this study is depicted in the model above (Fig 1), which illustrates the relationship between systematic strategic planning (SSP), sustainable performance (SP), and the mediating roles of strategic intent (SI) and strategic formulation (SF).

### 2.7. Theoretical framework

The theoretical foundation of this study is shown in the model above (Fig 1), which demonstrates the link among systematic strategic planning (SSP), sustainable performance (SP), and the mediating roles of strategic intent (SI) and strategic formulation (SF). While SSP is hypothesized to have a direct effect on SP, the theoretical framework acknowledges that much of this influence is transmitted indirectly via SI and SF. These mediating variables explain how strategic intent translates into cohesive planning, while strategic formulation ensures execution aligned with sustainability goals. This dual pathway—both direct and indirect—strengthens the logic of the proposed framework.

#### 2.7.1. Systematic strategic planning (SSP).

Systematic strategic planning serves as the essential framework in this situation. It signifies a methodical process that includes environmental assessment, goal establishment, and resource allocation to achieve organizational objectives. In the domain of manufacturing SMEs, SSP enables the effective management of operational challenges while aligning processes with sustainability goals. SSP is believed to directly influence sustainable performance by providing clarity and direction for long-term decision-making.

#### 2.7.2. Strategic intent (SI).

Strategic purpose denotes the organization’s commitment to a certain vision or goal. It embodies the aspirational aspect of strategy, fostering alignment and focus across all organizational levels. The mediating role of strategic intent in this framework suggests that SSP enhances sustainable performance by cultivating a shared understanding of objectives and creating a sense of purpose. Manufacturing SMEs benefit from a clear strategic objective by consistently pursuing sustainability and innovation.

#### 2.7.3. Strategic formulation (SF).

Strategic formulation involves transforming strategic objectives into actionable plans. This process involves identifying possibilities, evaluating risks, and devising specific strategies to achieve desired outcomes. The idea posits that SSP indirectly influences sustainable performance through strategy development, since effective planning yields actionable plans that improve operational efficiency, innovation, and environmental responsiveness.

#### 2.7.4. Sustainable performance (SP).

The ultimate objective of this paradigm is sustainable performance, encompassing economic, environmental, and social dimensions. For manufacturing SMEs, sustainable performance signifies the ability to maintain profitability, minimize environmental impact, and make a meaningful societal contribution. The notion asserts that SSP, along with the mediating effects of SI and SF, significantly enhances a SME’s ability to achieve enduring success.

## 3. Materials and methods

The target population for this study comprised senior managers, owners, and decision-makers of manufacturing SMEs registered with the Small and Medium Enterprises Development Authority (SMEDA) and relevant industry associations in Pakistan. These individuals were selected because they are directly involved in strategic decision-making processes that affect organizational sustainability performance.

A total of 600 questionnaires were distributed via email and in-person visits between January and April 2023. 410 valid responses were received, resulting in a response rate of 68.3%. After data screening and removing incomplete questionnaires, 410 cases were retained for analysis, ensuring consistency between the sample described in the methodology and the data presented in the results tables.

Participants were informed of the study’s purpose, anonymity was guaranteed, and their participation was voluntary. Informed consent was implied by the submission of completed surveys. Given the anonymous, non-sensitive nature of the data collected, the Institutional Review Board (IRB) of Hailey College of Banking & Finance waived formal ethical approval requirements, as noted in the ethical statement.

The study employed validated multi-item scales for all four constructs. SSP was measured using seven items adapted from [89], capturing dimensions such as strategic analysis, formulation, implementation, and monitoring. SP was assessed using nine items from [87,88], covering economic, social, and environmental outcomes. SI and SF were measured using four and three items respectively, derived from [90]. These items captured clarity of vision, commitment to long-term goals (for SI), and formulation processes such as SWOT analysis and action planning (for SF). All items were measured on a 5-point Likert scale. The questionnaire underwent expert review to ensure content validity.

Data were analyzed using Structural Equation Modeling (SEM) via AMOS 28 software. SEM was chosen due to its suitability for exploratory research and for testing complex models with multiple mediators. The analysis followed a two-step approach:

1. **Measurement Model Assessment:** Evaluated reliability (Cronbach’s alpha, composite reliability), convergent validity (Average Variance Extracted, AVE), and discriminant validity (Fornell-Larcker criterion).
2. **Structural Model Assessment:** Examined path coefficients, R² values, effect sizes (f²), and predictive relevance (Q²). Mediating effects of SI and SF were assessed through the bootstrapping technique.

## 4. Results

In demographic analysis (Table 1), we came to know the information about the study like gender, age marital status, experience and company size. The information is gathered through questionnaire and respective detail is shown in the table.

**Table 1:** Demographical characteristics of respondents.

Items			
Job Tenure	1-10 year	125	30.5
	11-20 year	194	47.3
	21-30 year	65	15.9
	31-40 year	26	6.3
	Total	410	100.0
Marital Status	non married	130	31.7
	married	280	68.3
	Total	410	100.0
Gender	female	256	62.4
	male	154	37.6
	Total	410	100.0
Age	25-30	56	13.7
	31-35	67	16.3
	36-40	124	30.2
	41-45	84	20.5
	46-50	39	9.5
	51-55	27	6.6
	56-60	13	3.2
	Total	410	100.0

The study sample encompasses various service sectors within Pakistan. SPSS was utilized to calculate frequencies and percentages for gender, designation, total experience, and type of organization. The respondent pool consists largely of young professionals with considerable experience in well-established organizations.

### 4.1. Data normality analysis

Table 2 displays the results of the data normality analysis. According to Bulmer (1979), skewness values should fall between −1 and +1 to indicate a normal distribution. Similarly, MacGillivary and Balandan established a range of −3 to +3 for kurtosis. As shown in Table 2, all skewness and kurtosis values are within these acceptable ranges, suggesting that the data is normally distributed and suitable for further analysis.

**Table 2:** Data skewness, mean and kurtosis.

Variables	Mean	St. Deviation	Skewness	Kurtosis
Systematic Strategic Planning	3.8146	.63793	−.740	.084
Strategic Performance	4.0195	.56824	−.855	.460
Strategic Intent	3.7354	.75988	−.754	.443
Strategic Formulation	3.8301	.77632	−.846	.575

### 4.2. Reliability analysis

Reliability analysis is crucial for assessing the consistency of the research instrument and the included items. This analysis helps identify and exclude problematic items before applying further statistical tests. A Cronbach’s alpha of 0.7 or higher is generally considered acceptable, with values above 0.8 indicating good reliability and values above 0.9 indicating excellent reliability. As shown in Table 3, all variables demonstrate Cronbach’s alpha values exceeding 0.7, suggesting good reliability. The overall reliability of the instrument is also above 0.7, indicating acceptable consistency and internal validity.

**Table 3:** Reliability Analysis.

Variable		No of items
Systematic Strategic Planning	**0.743**	**07**
Strategic Performance	**0.773**	**09**
Strategic Intent	**0.708**	**04**
Strategic Formulation	**0.725**	**03**
Over all reliability	**0.881**	**23**

### 4.3. Correlation analysis

Correlation analysis, presented in Table 4, examines the relationships between study variables. Correlation coefficients range from 0 to 1, with 1 indicating a strong positive relationship and 0 indicating no correlation. A significance level of p < .001 or p < .005 is typically considered statistically significant. The analysis reveals that all variables exhibit significant correlations at the 1% significance level.

**Table 4:** Correlation Analysis.

Items	SSP	SP	SI	SF
Systematic Strategic Planning	1			
Strategic Performance	.576^**^	1		
Strategic Intent	.408^**^	.544^**^	1	
Strategic Formulation	.410^**^	.571^**^	.437^**^	1

** Correlation is significant at the 0.01 level (2-tailed).

* Correlation is significant at the 0.05 level (2-tailed).

### 4.4. Structural equation model (SEM)

**Fig 2:**
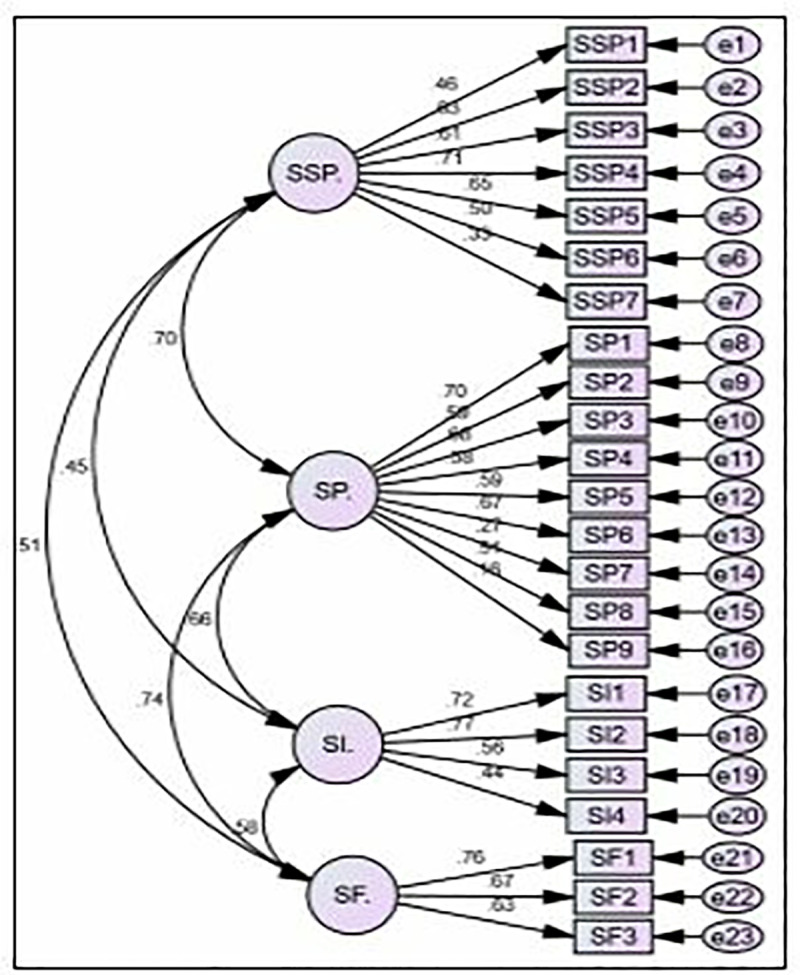
CFA Model Validates measurement constructs for SSP, SI, SF, and SP.

Structural equation modeling (SEM) was employed to analyze the relationships between variables, allowing for the simultaneous testing of multiple relationships and incorporating latent variables. SEM is useful for both exploratory and confirmatory analysis. Specifically, this study utilized confirmatory factor analysis (CFA) with AMOS 28 to assess the measurement model [91]. Figures were generated for each individual factor (Fig 2). Key fit indices were assessed (Table 5) to determine the adequacy of the model fit. A CFI value close to 1.00 indicates a good fit [92,93]. An RMSEA value below 0.08 suggests acceptable fit, and values below 0.06 indicate good fit [94]. GFI and AGFI values above 0.90 are generally considered indicative of good model fit. In this study, the CFI value is 1.00, the RMSEA value is 0.07, GFI is 0.999, and AGFI is 0.989, suggesting a well-fitting and acceptable model according to [92].

**Table 5:** Fitness Summary.

Model	Hypothesized	Thresholds
CMIN/DF	2.240	< 3
RMR	0.045	Closer to 0
GFI	0.900	≥ 0.9
AGFI	0.901	≥0.8
CFI	0.915	≥0.9
RMSEA	0.042	<0.08

#### 4.4.1. SI mediates between the SSP and SP.

**Fig 3:**
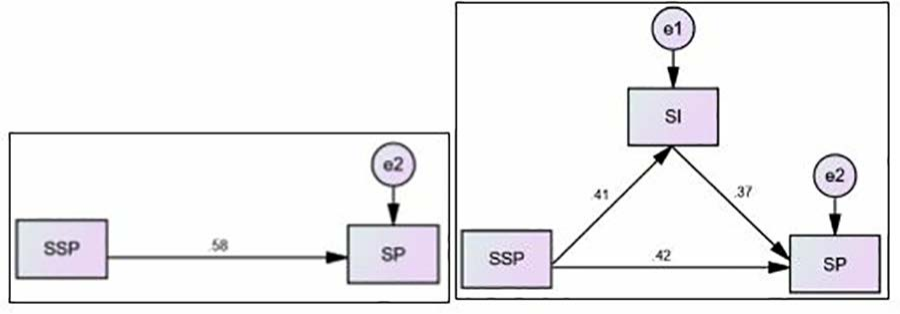
Path Model SSP→SI→SP.

Table 6 and Fig 3a, b examine the mediating role of SI in the relationship between SSP (independent variable) and SP (dependent variable). The direct effect of SSP on SP, without considering mediation, is β = 0.465 and p = 0.001. With SI included as a mediator, the direct effect of SSP on SP is reduced to β = .280 and p = 0.001. This suggests that SI partially mediates the relationship. The indirect effect of SSP on SP through SI (i.e., the mediation effect) is β = 0.215 and p = 0.001.

**Table 6:** Interpretation for Med. (SI Mediation Analysis).

Hypothesis	Dβ W/O Med.	Dβ with Med.	Iβ	Med. Type
**SSP→SI→SP**	β = 0.465 p = 0.001	β = .280 p = 0.001	β = 0.215 p = 0.001	Partial Mediation

#### 4.4.2. SF mediates between the SSP and SP.

**Fig 4:**
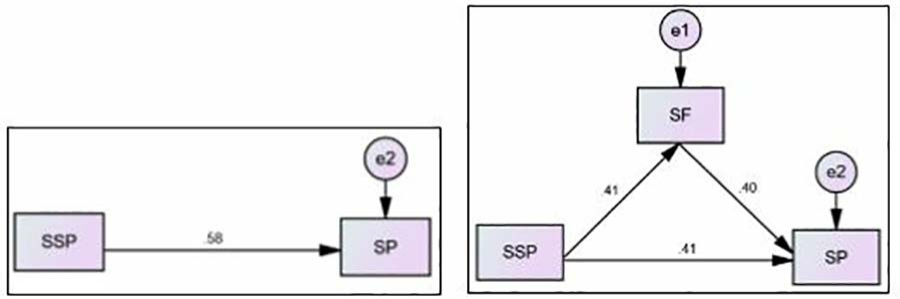
Path Model SSP→SF→SP.

Table 7 and Fig 4a, b examine the mediating role of SF in the relationship between SSP (independent variable) and SP (dependent variable). The direct effect of SSP on SP, without considering mediation, is β = 0.410 and p = 0.001. With SF included as a mediator, the direct effect of SSP on SP is reduced to β = .210 and p = 0.001. This indicates that SF partially mediates the relationship. The indirect effect of SSP on SP through SF (i.e., the mediation effect) is β = 0.205 and p = 0.001.

**Table 7:** Interpretation for Med. (SI Mediation Analysis).

Hypothesis	Dβ W/O Med.	Dβ with Med.	Iβ	Med. Type
**SSP→SF→SP**	β = 0.410 p = 0.001	β = .210 p = 0.001	β = 0.205 p = 0.001	Partial Mediation

## 5. Discussion

This study identified SSP and SBI as essential elements in improving the sustainable performance (SP) of manufacturing SMEs. The direct effect of SSP on the SP of manufacturing SMEs in Pakistan is significant and positive, shown by a B-value of 0.4 for the relationship between the independent variable SSP and the dependent variable SP, surpassing 0.1. The T-Statistics and p-values indicate that the relationships between SSP and SP are significant, since the p-values are below 0.05 and the T-Statistics surpass 1.96, confirming the significance of hypothesis H1. This result is consistent with other recommendations and prior studies indicating a substantial and advantageous effect of strategic planning on performance and sustainability [95–97]. Moreover, the study indicates that the T-Statistics and p-values reveal substantial correlations between SSP > SI > SP and SSP > SF > SP, as the p-values are below 0.05 and the T-Statistics surpass 1.96. This signifies the significance of hypotheses H2 and H3; empirical evidence supports that strategic intent (SI) and strategic formulation (SF) positively affect sustainable performance (SP) in Pakistani manufacturing SMEs, hence confirming hypotheses H2 and H3. This conclusion corresponds with the findings of previous research that have recognized a substantial and advantageous impact of strategic innovation on company performance and sustainability [98]. The findings corroborate the TBL and RBV theories, suggesting that executives may employ systematic strategic planning (SSP) in their operations to get a competitive edge and secure their company’s success in attaining economically, ecologically, and socially sustainable performance. The results demonstrate that thorough strategic planning, including strategic intent and formulation, is essential for improving organizational performance and motivating SMEs to pursue innovative and aggressive initiatives.

Although the findings confirm a significant relationship between SSP and SP, it is important to note that cross-sectional data may give rise to potential endogeneity concerns. Specifically, firms with strong sustainable performance may also be more inclined to engage in strategic planning, creating reverse causality. To address this concern, the study has included SI and SF as mediators, which help reduce omitted variable bias and enhance the explanation of causal mechanisms. Moreover, the statistical model was assessed for multicollinearity and indirect effects to improve the robustness of inferences.

### 5.1. Theoretical contribution

This study advances the understanding of strategic planning and sustainability in SMEs by introducing SI and SF as mediators. It extends TBL and RBV theory by offering a comprehensive framework that explains how SSP translates into improved sustainable performance, specifically addressing the economic, environmental, and social dimensions of sustainability. The research fills a gap by focusing on manufacturing SMEs in Pakistan—a setting characterized by volatile political and economic conditions—providing insights often overlooked in previous studies [99].

This study contributes to the literature by expanding the comprehension of sustainable performance in manufacturing SMEs within developing nations experiencing political and economic uncertainty. Thus, the research findings demonstrate that SSP and SBI are vital management tools for organizations operating in highly competitive and unstable environments. The issue of this study may be analyzed from several theoretical perspectives due to the scarcity of empirical research about the link among SSP, SI, SF, and SP in Pakistani manufacturing SMEs. Theories encompass sustainable performance theory, Triple Bottom Line (TBL) theory, and Resource-Based View (RBV). The study utilizes the Triple Bottom Line and Resource-Based View as theoretical frameworks to analyze the research topic. The ramifications and significance of the theory are integral to the subject under research. Thus, this essay aims to address the theoretical gap in the existing research. The literature thoroughly investigates the effectiveness and impact of strategic planning on corporate performance in large enterprises, consulting firms, and significant manufacturers under a stable political environment [5,100]. Nonetheless, several studies were undertaken in various developing nations marked by a dynamic economic landscape. This study investigates the relationship between systematic strategic planning (SSP), strategic intent (SI), strategic formulation (SF), and sustainable performance (SP) in the context of Pakistani manufacturing SMEs, which are currently confronting an unstable and dynamic environment that hinders their growth and success.

Furthermore, previous studies investigating the correlation between strategic planning and organizational performance have mostly concentrated on businesses in developed and emerging countries [17]. This research is one of the first studies to model and analytically investigate the link between SSP and SP, with SI and SF serving as mediators in the context of developing nations. In contrast, less study has explored the many aspects of strategic planning dynamism, especially in turbulent environments, as noted by [101]. This study seeks to concurrently examine the many elements of systematic strategic planning that were overlooked in previous studies on rising nations. This study augments the current knowledge by presenting a conceptual framework that analyzes the relationship between independent variables, such as SSP, and mediating variables, including SI, SF, and SP as dependent variables. This research investigates the influence of strategic intent categories and strategic formulations on the sustainable performance of manufacturing SMEs in a developing country. This research, in contrast to prior studies, emphasizes the necessity of expanding the assessment of manufacturing SMEs’ performance beyond financial indicators to encompass non-financial indicators, integrating economic, social, and ecological metrics designated as SP by this study [102].

### 5.2. Managerial and practical ramifications

Pakistan is widely seen as an unstable nation due to its political conditions; thus, its foreign direct investment is restricted and insufficiently focused on manufacturing industries [103]. Moreover, major firms such as Google, Intel, Facebook, Burger King, and Starbucks do not have any branches in Pakistan [104]. Considering that most manufacturing firms in Pakistan are small and medium-sized companies (SMEs) with constrained resources, there is a paucity of understanding on the impact of strategic factors on sustained business performance. Moreover, SMEs generally devote limited resources to strategic planning, emphasizing short-term outcomes over long-term objectives [54–56,78]. As a result, Pakistani manufacturing firms face both external and internal obstacles that hinder their operational efficiency and sustainability. The challenges encompass a deficiency in strategic direction, inadequate governance and institutional frameworks, shortcomings in managerial, operational, and production environments, insufficient marketing and technological infrastructures, limited research and development initiatives, and the incapacity of many manufacturers to establish quality control systems and comply with international standards such as ISO 9001, ISO 50001, ISO 14001 for Environmental Management Systems, and Six Sigma, among others. Numerous challenges may be mitigated with thorough strategy planning [38,62]. In contrast, local investors refrain from long-term planning owing to unforeseen situations. Similarly, several industrial companies are family-owned businesses where the owners are reluctant to dedicate a portion of their sales or earnings to formalizing and organizing their operations [76]. As a result, corporations hesitate to expand their operations and investments due to an absence of strategic vision and uncertainty in the business climate. The research advises the government to provide training assistance for innovation to manufacturing SMEs and to encourage creative endeavors. This claim assumes that only skilled and trained human capital can develop strategic objectives and formulate strategies. This research has policy ramifications for improving strategic intent and development inside Pakistani manufacturing SMEs. This paper outlines eight distinct ways for manufacturing SMEs in Pakistan to innovate strategically; nevertheless, the execution of all suggested innovation types may be unfeasible, especially in a developing nation such as Pakistan. The importance of methodical strategic planning has increased as a vital factor in a business’s overall performance. The impact of diverse strategic innovation initiatives on performance requires more examination. Similarly, strategic business innovation and its impact on sustainable performance are fraught with uncertainty. In developing countries, especially in the Arab World, little attention has been given to examining this connection, making it a noteworthy topic for further research. From a decision-making standpoint, the findings offer a structured roadmap for SME managers. First, implementing SSP practices can provide clarity in uncertain environments and reduce decision-making errors. Second, articulating a strong strategic intent ensures all stakeholders align their efforts with long-term sustainability goals. Third, formalizing strategy formulation empowers managers to translate vision into coherent actions. SME leaders are encouraged to institutionalize planning cycles and data-driven decision processes. Policymakers, too, can play a crucial role by offering capacity-building programs to enhance SMEs’ planning capabilities. These practical insights make the findings actionable and relevant for real-world application.

### 5.3. Future research

Despite its contributions, this study has several limitations. First, it focuses on manufacturing SMEs in Pakistan, which may limit the generalizability of findings to other industries or regions. Second, the cross-sectional design prevents assessment of long-term impacts. Future research could explore longitudinal studies to examine the sustained impact of SSP on performance over time. Comparative studies across different countries and sectors would also enhance generalizability. Additionally, incorporating qualitative approaches could provide deeper insights into how strategic planning processes are operationalized within SMEs. Future studies should consider longitudinal or experimental research designs to better address endogeneity. Using instrumental variables or panel data could provide deeper insights into the causal relationship between SSP and sustainable performance.

## 6. Conclusion

In conclusion, this study provides actionable recommendations for owners and managers to mitigate risks and capitalize on opportunities through strategic planning, innovation, human capital investment, and stakeholder engagement. The findings are highly relevant for policymakers and managers in manufacturing and medium-sized firms. A key takeaway is that systematic strategic planning (SSP) should be viewed as a critical commitment, not just a compliance exercise. Its positive influence on factors like decision-making processes directly contributes to planning success. SMEs should embrace rational, data-driven decision-making within a structured strategic framework to achieve improved economic, environmental, and social outcomes. By prioritizing a scientific approach over emotional impulses, managers can fully realize the benefits of SSP, including enhanced efficiency, increased production, and improved sustainability.

Furthermore, the study highlights the interconnectedness of strategic plan formulation, execution, and progress assessment. Optimal planning results are often achieved through the simultaneous implementation of SSP and robust decision-making procedures. Pakistani manufacturing and medium-sized firms can leverage the outlined systematic strategic planning methodology to better navigate volatile economic conditions. Integrating SSP with the enhancement of organizational skills and decision-making processes can bolster their crisis management capabilities, leading to effective crisis management strategies and timely strategic decisions. As manufacturing and medium-sized companies grow and face increasingly complex sustainability challenges, SSP must ensure efficient communication of corporate goals and plans throughout the organization, fostering greater transparency and organization. Decision-makers in these enterprises can utilize the study’s insights on resource management to drive sustainable performance. Ultimately, senior management within SMEs must recognize the vital role of methodical strategic planning in achieving enhanced sustainable performance.

Finally, cultivating a culture of innovation within SMEs is crucial, as strategic formulation and intent are essential for survival and act as a significant source of competitive advantage. SMEs must recognize the potential of strategic purpose and value enhancement through rigorous strategic planning methodologies. This study’s examination of the impact of SSP on the long-term performance of Pakistani SMEs, along with the mediating roles of strategic planning and formulation, suggests that adhering to these recommendations will improve management and operations. The study’s applicability extends to both public and private enterprises, and future research could explore the application of these strategies across diverse sectors like banking, insurance, and telecommunications. The academic community will benefit from understanding how SSP, including strategic intent and strategy formulation, enhances the sustainable performance of the manufacturing sector, particularly within SMEs – an area often overlooked in prior research. In today’s rapidly changing business environment, an organization’s operational capabilities and resources are increasingly strained, influencing management and employee behavior. Combining both disciplines will yield new knowledge that is especially beneficial for SMEs. This study fills a gap in the literature by focusing on the unique context of production and medium-sized firms and the interconnectedness of these elements, offering valuable insights for managers, specialists, and decision-makers in both public and commercial sectors.

## Abbreviations

SP: *Sustainable Performance*
SSP: *Systematic Strategic Planning*
SI: *Strategic Intent*
SF: *Strategic Formulation*
SMEs: *Small and Medium Enterprises*
SDGs: *Strategic Development Goals.*
