# Expression of Concern: Strategic planning as a catalyst for sustainability: A mediated model of strategic intent and formulation in manufacturing SMEs

**DOI:** 10.1371/journal.pone.0354302

**Published:** 2026-07-22

**Authors:** The PLOS One Editors

After this article [1] was published, the following concerns were noted:

- The article cited as Reference 94 was retracted before [1] was published.
- The article cited as Reference 44 was retracted after [1] was published.
- A lack of supporting citations for statements in the Introduction section.
- The articles cited as References 3, 4, 5, 14, 36, and 70 do not appear to support the corresponding cited statements.

In addition, the *PLOS One* Editors have concerns about compliance with the PLOS Authorship policy.

In light of the cumulative issues, the *PLOS One* Editors issue this Expression of Concern. Readers are advised to interpret the article [1] with caution.

## References

[1] Mehta AM, Qazi SZ, Haque R, S Senathirajah ARb, Baig W, Sajjad R, et al. Strategic planning as a catalyst for sustainability: A mediated model of strategic intent and formulation in manufacturing SMEs. PLoS One. 2025;20(6):e0325887. doi: 10.1371/journal.pone.0325887 40493573 PMC12151430

